# Defining, conceptualizing and evaluating pragmatic qualities of quantitative instruments measuring implementation determinants and outcomes: a scoping and critical review of the literature and recommendations for future research

**DOI:** 10.1093/tbm/ibac064

**Published:** 2022-11-01

**Authors:** Louise Hull, Richard Boulton, Fiona Jones, Annette Boaz, Nick Sevdalis

**Affiliations:** Centre for Implementation Science, Health Service and Population Research Department, King’s College London, London, UK; Centre for Health and Social Care, St George’s, University of London and Kingston University, UK; Centre for Health and Social Care, St George’s, University of London and Kingston University, UK; Faculty of Public Health & Policy, London School of Hygiene & Tropical Medicine, London, UK; Centre for Implementation Science, Health Service and Population Research Department, King’s College London, London, UK

**Keywords:** Implementation science, Implementation measures, Implementation outcomes, Implementation determinants, Measurement, Pragmatic measures

## Abstract

The pragmatic (i.e., practical) quality of quantitative implementation measures has received increased attention in the implementation science literature in recent years. Implementation measures that are judged to be pragmatic by implementation stakeholders are thought to be more likely to be applied in research and practice. Despite the need for pragmatic implementation measures, ambiguity and uncertainty regarding what constitutes a pragmatic measure remains. This study sought to identify and critically appraise the published literature to understand (i) how pragmatism is defined as a measurement construct/quality of implementation determinants and outcome instruments; (ii) how pragmatic qualities of instruments are evaluated; (iii) identify key gaps and limitations of the current evidence-base and (iv) identify recommendations for future research. We conducted a scoping review of the literature also employing methods of critical review. PubMed and PsycINFO databases, using the OVID interface, were searched for relevant articles published between January 2010 and September 2020. Articles that contained a definition and/or described characteristics of “pragmatism” as a measurement construct of quantitative implementation outcomes (as defined by Proctor’s Implementation Outcomes taxonomy) and/or implementation determinants were eligible for inclusion. Nine articles met inclusion criteria. A degree of overlap in definitions and terms used to describe the pragmatic qualities of quantitative implementation determinant and outcome instruments were found. The most frequently cited descriptors of pragmatism were “not burdensome”, “brief”, “reliable”, “valid” and “sensitive to change”. 3 of the 9 included articles involved international implementation stakeholders in defining and conceptualizing pragmatism and employed specific methods to do so, including a systematic literature review, stakeholder interviews, concept mapping, and a Delphi process. All other articles defined pragmatism, with or without citing relevant literature. One article objectively assessed the pragmatic qualities, above and beyond the psychometric qualities, of implementation measures, using the Psychometric and Pragmatic Evidence Rating Scale (PAPERS). The evidence base within the implementation instrumentation literature on what pragmatism is and how it might be assessed is limited. Some of the research identified in the review provides a strong foundation to build upon, by testing its applicability in other settings (including healthcare areas and countries) and among a more diverse group of stakeholders. We discuss directions for further development of the concept of pragmatism relating to the measurement of implementation determinants and outcomes.

## Teaser

We explore how pragmatic (i.e., practical) qualities of quantitative implementation measures are defined, conceptualised and evaluated within implementation science. In doing so, we identify several gaps and limitations of the current evidence-base.

**Implications**

**Practice:** The availability of psychometrically robust (i.e., reliable and valid) as well as pragmatic (i.e., practical) implementation measures is vital to plan and evaluate implementation efforts.

**Policy:** Application of psychometrically strong and pragmatic quantitative implementation measures can help policy-makers monitor and adjust implementation efforts for interventions or programs.

**Research:** Future research should engage a more diverse group of implementation stakeholders in defining and evaluating pragmatic qualities of implementation measures.

## Introduction

Over the past decade, discussions relating to pragmatic implementation research and practice, and specific elements and design considerations of implementation research and practice, have increased in the implementation science literature. Perhaps not surprisingly, pragmatic implementation study design, and in particular, pragmatic trial design has received the most attention to date and tools such as the PRagmatic Explanatory Continuum Indicator Summary (PRECIS) tool [1], have been developed and recently refined (PRECIS-2) [2]. More recently, discussions of pragmatism in the implementation science literature have expanded in focus and typically pragmatism has been discussed in relation to a specific element/design considerations of implementation research and practice. For example, the practicality of implementation strategies [3], the pragmatic application of implementation frameworks [4], pragmatic approaches to analyzing qualitative implementation data [5], and pragmatic qualities of quantitative implementation measures [6, 7].

Implementation measures considered not to be pragmatic are problematic for several reasons. First, implementation stakeholders are unlikely to use implementation measure that are not considered pragmatic, even if they are psychometrically strong [7, 8]. Second, without the time and expertise to develop and/or adapt existing measures, the selection of lengthy measures (i.e., unpragmatic), may create a situation in which implementation stakeholders are forced to evaluate only the outcomes and determinants considered critical to implementation, rather than the full spectrum of outcomes and determinants of interest. Ultimately limiting the study and understanding of factors facilitating and impeding implementation. Third, it is plausible that lack of pragmatic measures might lead implementation stakeholders to develop measures, referred to as “home-grown” instruments [9], and/or adapt existing instruments. This is a cause for concern as without specialist expertise in instrument development, the development and/or adaptation of existing instruments is likely to lead to the development and application of implementation measures that are neither valid nor reliable.

The lack of pragmatic (i.e., practical) quantitative implementation measures has been identified as one of several measurement challenges hampering the advancement of implementation research and practice [9]. Martinez et al. [9] put forth four broad categories of instrument practicality and associated recommendations that instrument developers may wish to bear in mind; Costs (avoid the proprietary and commercialization of instruments); Accessibility (share instruments in existing repositories and/or publications); Length (limit number of constructs and items); and Language (avoid complex and ambiguous language). Other measurement challenges include the use of instruments without established psychometric properties, and the use of `home-grown’ and/or adapted instruments [9]. Recommendations to limit the impact of these challenges on the field have been proposed [9]. Over the past few years, there has been a consistent effort in the implementation science literature to address the aforementioned challenges, with early efforts focusing on identifying and appraising the psychometric qualities of quantitative instruments developed to evaluate implementation determinants and outcomes [10–12]. Several systematic reviews have been conducted to identify and appraise the psychometric and methodological quality of quantitative implementation measurement instruments developed and validated for use in mental or behavioral health [10], physical health [11], and public health and community settings [12], for the evaluation of a wide range of implementation determinants and outcomes. These reviews have typically found that very few instruments developed and applied in the field have had their psychometrical properties rigorously evaluated [10–12]. Instruments that are psychometrically strong are essential to advancing our understanding of mechanisms, mediators, moderators and determinants of implementation, which is critical to moving the field forward [13].

More recently, the pragmatic quality of quantitative implementation instruments has also received some attention—which has been described as a necessary and highly desirable attribute of implementation instruments [7]. Despite such efforts, ambiguity and uncertainty regarding what constitutes a pragmatic implementation measure remains. Almost a decade ago, Glasgow et al. proposed a set of required criteria for pragmatic measures of treatment outcomes, including: “importance to stakeholders”; “low respondent and staff burden”, “actionable and sensitive to change”; as well as additional recommended criteria, including: “broadness of”; “serves as a benchmark”; “unlikely to cause harm”; “psychometrically strong”; and “related to theory or model” [14]. However, whether pragmatic qualities can be transferred from treatment outcome measurement to implementation measurement is unclear. Since this initial discussion of pragmatism, a growing body of literature has emerged to engage with these issues relating specifically to implementation measures [6–8]. The reasoning behind the drive for psychometrically strong as well as pragmatic measures is that implementation measures deemed to be pragmatic are more likely to used, enriching our understanding of factors facilitating and impeding implementation and ultimately improving the implementation and sustained use of evidence-based practices. Please delete the following text: that are not pragmatic will only be of value and limited to use within the context of well-resourced research settings (e.g., research-funded largescale implementation or hybrid trials), further increasing the gap between research and practice. Pragmatic implementation measures are critical in low-resource settings, where resources for data collection are typically more limited. But even in high-resource settings, where resources are overall better, in our experience of designing implementation trials and studies within healthcare settings in the UK [15–17], there is reluctance and resistance amongst implementation stakeholders to apply measures too burdensome and too lengthy (i.e., not pragmatic).

It has further been argued that the availability of pragmatic implementation instruments is hampered by the fact that instrument developers do not traditionally approach the tool development process with both psychometric and pragmatic considerations concurrently in mind [18]. Inspection of the available evidence on implementation instruments suggests that instrument developers have focused on establishing the psychometric qualities of implementation measures (reliability, validity) far more than on pragmatic qualities. This is evident in the number of systematic reviews that have been published that have sought to identify and appraise the psychometric quality of implementation outcome instruments. More recently published systematic reviews have sought to appraise the pragmatic qualities, as well as the psychometric quality of implementation measures [19] and efforts to develop pragmatic measures that are also psychometrically strong have taken place [20]. Furthermore, notable recent efforts have also endeavored to define, operationalize and appraise the pragmatic qualities of implementation measures, shifting from simpler measures of pragmatism, such as number of items in a measure [10, 11], to more comprehensive and stakeholder-driven conceptualization and appraisal of pragmatism [6, 8].

Specifically, there seems to be confusion and a lack of clarity or distinction between psychometric and pragmatic qualities in the implementation science literature, with the terms used to describe different constructs/qualities of implementation measures and also psychometric quality described as a pragmatic quality. One concern is how pragmatism can be further conceptualized without creating further theoretical abstraction. For example, can an instrument be psychometrically strong and pragmatic at the same time? Or is a trade-off between psychometric strength and pragmatic quality inevitable? Such questions require clearer treatment. Here we argue that more clarity is needed in how pragmatism is defined and conceptualized within the field; how it impacts the instrument development process and procedures; and the way in which the pragmatic qualities of an instrument are evaluated and reported in implementation studies.

This review aims to contribute to the conceptual clarification of pragmatism, as a measurement construct, within implementation science. We specifically aimed to identify and critically appraise published literature that 1) seeks to define/operationalize pragmatism as a measurement construct/quality of quantitative implementation determinants and outcome instruments and 2) identifies how pragmatic qualities of implementation determinant and outcome instruments are evaluated. In doing so, we also sought to identify key gaps and limitations of the current evidence-base in respect to the definition, operationalization and appraisal of pragmatism.

## Methods

### Review framework

We conducted a scoping review of the literature, incorporating aspects of a critical review. Our definition of a scoping review is taken from Grant and Scott’s typology of reviews [21]: i.e., a scoping review “aims to identify the nature and extent of research evidence […] And characterizes quantity and quality of literature perhaps by study design and other key features.”, whilst not incorporating the extensive synthesis and appraisal measures of other reviews [21]. Furthermore, according to the aims and objectives of the review, we also sought to incorporate aspects of a critical review “to ‘take stock’ and evaluate what is of value from the previous body of work. In the context of this review, we sought to establish and critically appraise the methodologies utilized and the stakeholders that have been involved in defining and conceptualizing pragmatism, to evaluate the value of previous research and identify future research recommendations. It may also attempt to resolve competing schools of thought. As such, it may provide a ‘launch pad’ for a new phase of conceptual development and subsequent ‘testing’” [21]. This aspect of the review process was incorporated in the way in which the extracted data was interpreted and analyzed, and the aspects focused upon. For example, we were interested in critically examining the diversity and number of implementation stakeholders that have been involved in defining/conceptualizing the pragmatic quality of implementation measures. And at the same time, we were interested in critically identifying the implementation stakeholder groups that have not been involved in defining/conceptualizing pragmatism to-date. Similarly, we were interested in identifying the methods employed to define/conceptualize pragmatism and critically appraising, and identifying associated limitations of, the methods used. In identifying the limitations of the current evidence base and making several recommendations for future research, our review identifies several fruitful directions for the next phase of conceptual work aiming to define, conceptualize and evaluate the pragmatic qualities of implementation determinant and outcome measures.

Our first step was to identify works already displaying some overview of the field. The first author identified 5 key articles [6–8, 14, 22] that informed the search strategy and data extraction form. These articles were also used as a means of checking the sensitivity of the search. In particular, we sought to narrow the focus of the systematic review conducted by Stanick et al. [6] on pragmatic measure constructs to begin to frame our research criteria and focus. This article was chosen as, to the best of our knowledge, represents the only literature review of the pragmatic construct to date.

### Search strategy

PubMed and PsycINFO databases were searched, using the OVID interface, for relevant articles published between January 2010 and September 2020. The final search was conducted on 28th September 2020. The search terms and strategy were informed by the systematic review that sought to identify synonyms, descriptors, or dimensions, of the “pragmatic” construct, conducted by Stanick et al. [6]. (See Table 1 for search terms and strategy, including truncation (e.g., instrument*) and Boolean operators used (i.e., OR, AND, NOT)). The search was restricted to title and abstract. The date restriction (i.e., articles published after 2010) was applied as pragmatic measurement has only recently gained attention in the implementation science literature.

**Table 1.** Search terms and strategy

Level	Search terms
1	Pragmatic AND
2	Assessment* OR measure* OR instrument* OR questionnaire* OR survey* AND
3	implementation NOT
4	Language

Note: The “NOT language” was used (in agreement with the Stanick et al. systematic review) to exclude the substantial number of articles on the subject of “pragmatic language disorder” that are not relevant to this review. The search was restricted to titles and abstracts, articles published in English and published from 2010 onwards. *Search terms in italics represent additional terms used in the current review in addition to the term used by Stanick et al.[6].

Although we applied a very similar search strategy to that employed by Stanick et al., our review sought to identify articles that specifically focused on defining and identifying pragmatic qualities of quantitative implementation determinants and outcome measures. In contrast, Stanick et al.’s [6] approach was broader and did not exclude articles based on scientific discipline or type of outcomes.

### Inclusion criteria

1. Articles that contain a definition and/or describe characteristics of “pragmatism” as a measurement construct of quantitative implementation outcomes (as defined by Proctor’s Implementation Outcomes taxonomy) and/or implementation determinants
2. Articles relevant to health (physical, mental and behavioural) and social care
3. Peer-reviewed journal articles

### Exclusion criteria

1. Articles describing an implementation determinant or outcome instrument as “pragmatic” without defining and/or describing characteristics of pragmatic
2. Articles describing pragmatic study designs
3. Articles describing the pragmatic application of implementation theories, frameworks and models
4. Articles describing the pragmatic implementation of an innovation, intervention or service
5. Articles describing pragmatic implementation strategies
6. Articles describing a pragmatic innovation, intervention and/or service
7. Articles describing research protocols

We decided to focus on articles that defined and/or described characteristics of “pragmatic” as a measurement construct of quantitative implementation determinant and implementation outcomes instruments. In the context of this review, we defined implementation outcomes “as the effects of deliberate and purposive actions to implement new treatments, practices, and services” [22] and implementation determinants as “factors believed or empirically shown to influence implementation outcomes” [23]. The decision to focus on implementation outcomes and determinants was made in the interests of ensuring that the articles included were not too broad in measurement focus and would allow us to contribute to the conceptual clarity of pragmatism within implementation science.

### Screening of articles

Articles were screened for relevance at title and abstract stage by the first author. The second author independently screened 20% of the articles at title and abstract stage to ensure accuracy in screening. The results of both authors screening were then compared, discrepancies identified and discussed, and the inclusion/exclusion criteria further refined. Articles that warranted screening at full-text stage, were split equally so that the first and second author reviewed and extracted data from 50% of the articles. The first and second author reviewed each other’s inclusion/exclusion decisions and data extraction for accuracy and completeness. Discrepancies at each stage of screening were resolved through discussion between the first and second author until consensus was reached.

### Data extraction

A standardized data extraction form was developed (see Supplementary File 1 for data extraction form). We extracted the following data from articles that met our inclusion criteria: lead author and year of publication; study type (i.e., empirical or theoretical) and country in which the study was conducted; implementation determinant(s) and/or outcome(s) of interest/focus (if applicable) and associated instrument(s) (if applicable); definition of pragmatism including associated descriptors; method(s) used to define/operationalize pragmatism as a measurement construct (in applicable); stakeholder groups involved in defining/conceptualizing pragmatism; details of whether pragmatism was assessed and how; and limitations identified by authors.

## Results

After duplicates were removed and date and language restrictions were applied, the search retrieved 731 articles. 680 articles were excluded at title and abstract stage, resulting in 52 full-text articles assessed for eligibility. A further 41 articles were excluded at full-text stage, resulting in 9 articles meeting inclusion criteria [6–8, 24–29].

### Study type and setting (country)

Eight of the 9 articles included were empirical studies [6–8, 24–28] and one was a theoretical article [29]. The one theoretical article included was written by authors based in the USA [29]. Of the 8 empirical studies included 4 were USA-based studies [24, 25, 27, 28] and 4 were international studies [6–8, 26]. The international studies included study participants/stakeholders and/or international stakeholder panels/advisory groups from the following countries:

- Study participants (*n* = 1256) from USA (*N* = 418) and Norway (*N* = 838) [26]
- Study participants/stakeholders (*n* = 26) consisting of stakeholders from USA (*n* = 25), Canada (*n* = 1) and the Netherlands (*n* = 1) [8]. Note: A total of 26 unique stakeholders participated in the Delphi study (see article for further details).
- Study participants/stakeholders (*n* = 7) consisting of stakeholders from USA (*n* = 5), Canada (*n* = 1) and the Netherlands (*n* = 1) [6].
- Study participants/stakeholders (*n* = 24) from USA (*n* = 23) and Canada (*n* = 1), including stakeholder panel (*N* = 4) consisting of stakeholders from USA (*n* = 3) and Canada (*n* = 1), and international advisory board (*n* = 9) consisting of advisors from Australia (*n* = 2), Canada (*n* = 2), Netherlands (*n* = 1), Sweden (*n* = 1), UK (*n* = 1), USA (*n* = 2) [7].

### Terms and phrases used to describe pragmatism

In what follows, we report how pragmatism was defined/conceptualized as a measurement construct of implementation determinant and implementation outcome instruments included in the review.

Table 2 presents the frequency count, across included articles, of descriptors and antonym descriptors of pragmatism used to define/conceptualize pragmatism, as a measurement construct of quantitative implementation determinant and/or implementation outcomes instruments. A degree of overlap, relating to the descriptors used to define pragmatism as a measurement construct of implementation determinant and outcome measures, was evident across the included articles. The most frequently cited descriptors of pragmatism were “not burdensome”, “brief”, “reliable”, “valid” and “sensitive to change”. Nine descriptors of pragmatism were only cited once across the included articles (i.e., these descriptors were unique to the article in question). These included, “practical”, “ability to be tailored”, “appropriate”, “length”, “important to stakeholder”, “can be used for benchmarking”, “has norms”, “salient to both stakeholders and researchers” and “has high-utility”. Four antonym descriptors of pragmatic were identified, including “time-consuming to administer”, “expensive to administer”, “proprietary” and “lengthy”.

**Table 2.** Frequency count of descriptors of pragmatism used across included articles

Descriptors of pragmatism	Frequency count across included articles (*n* = 9)
Not burdensome	7/9 [6–8, 25–27, 29]
Brief	6/9 [6–8, 24, 25, 29]
Reliable	5/9 [6–8, 24, 28]
Valid	5/9 [6–8, 24, 28]
Sensitive to change	5/9 [6, 7, 25, 26, 29]
Efficient	4/9 [6–8, 24]
Low Cost	4/9 [6–8, 29]
Feasible	4/9 [6–8, 29]
Broadly applicable	4/9 [25–27, 29]
Actionable	3/9 [6, 25, 29]
Acceptable	3/9 [6–8]
Easy	3/9 [6–8]
Compatible	3/9 [6–8]
Useful	3/9 [6–8]
Creates a low social desirability bias	3/9 [6–8]
Relevant	3/9 [6–8]
Offers relative advantage over existing methods	3/9 [6–8]
Applicable	3/9 [6–8]
Easy to interpret	3/9 [6–8]
Not wordy	3/9 [6–8]
Completed with ease	3/9 [6–8]
Uses accessible language	3/9 [6–8]
Informs decision making	3/9 [6–8]
Fits organizational activities	3/9 [6–8]
Unlikely to cause harm	2/9 [25, 26]
Psychometrically strong	2/9 [25, 26]
Related to theory/models of implementation	2/9 [25, 26]
Transparent	2/9 [6, 7]
Tied to reimbursement	2/9 [6, 7]
Focused	2/9 [6, 7]
The output of routine activities	2/9 [6, 7]
Non-duplicative	2/9 [6, 7]
Offers flexible administration time	2/9 [6, 7]
Easy to administer	2/9 [6, 7]
Requires no expertise	2/9 [6, 7]
Of low complexity	2/9 [6, 7]
Accessible by phone	2/9 [6, 7]
Intuitive	2/9 [6, 7]
Simple	2/9 [6, 7]
Easy to use	2/9 [6, 7]
Easy to score	2/9 [6, 7]
Automated scoring	2/9 [6, 7]
Compatible	2/9 [6, 7]
Leads to intervention or treatment plan	2/9 [6, 7]
Connects to clinical outcomes,	2/9 [6, 7]
Important to clinical care	2/9 [6, 7]
Reveals problems/issues in process or outcomes	2/9 [6, 7]
Informs adherence of fidelity	2/9 [6, 7]
Assesses organizational progress over time	2/9 [6, 7]
Meaningful	2/9 [6, 7]
Confirms efficacy of interventions	2 [6, 7]
Has a meaningful score distribution	2 [6, 7]
Optimizes patient care	2 [6, 7]
Informs clinical intervention selection	2 [6, 7]
Practical	1 [6]
Ability to be tailored	1 [25]
Appropriate	1 [8]
Length	1 [8]
Important to stakeholders	1 [26]
Can be used for benchmarking	1 [26]
Has norms	1 [26]
Salient to both stakeholders and researchers	1 [27]
Has high utility	1 [28]
**Antonyms of pragmatism**	
Time-consuming to administer	[7]
Expensive to administer	[7]
Proprietary	[6]
Lengthy	[6]

### Definition and conceptualization of pragmatism

In addition to calculating the frequency of descriptors of pragmatism across articles, we extracted the definitions and descriptors of pragmatism for each of the included articles, noted whether the definition was provided in relation to a specific implementation determinant and/or outcome instruments, and whether the authors cited relevant literature to support the definition/description of pragmatism. The results are presented in Table 3.

**Table 3.** Definitions and descriptors of pragmatism as a measurement construct of quantitative implementation determinants or implementation outcomes instruments

Authors	Implementation determinant and/or implementation outcome of interest	Definition, descriptors of pragmatism	Literature that authors cite to support definition/description of pragmatism
Aarons et al. 2016 [24]	Implementation leadership	*“pragmatic (i.e., brief, reliable, valid)”* “The ILS *(implementation determinant instrument)* is a brief, efficient, and pragmatic measure that can be administered quickly, typically taking no more than a few minutes to complete. This supports the pragmatic nature of the measure as it reduces the amount of time taken away from job duties for those completing the measure”	None
Battaglia et al. 2018 [29]	Pragmatism not conceptualised in relation to a specific implementation determinant or outcome	“By a pragmatic measure, we mean an assessment strategy that is feasible to use in busy real-world settings, is brief, low cost, actionable, produces rapid or immediate feedback, is not burdensome, yet still broadly applicable, and sensitive to change”	Glasgow et al. Pragmatic measures what they are and why we need them. Am J Prev Med. 2013;45(2):237–43 [14].
Moullin et al. 2017 [25]	Implementation intentions	“the movement toward pragmatic measures that are brief, have low burden, are sensitive to change, and have broad applicability suggests the need for a measure that can be tailored for any specific EBP or innovation that is being implemented” “making the scale more pragmatic by reducing the number of items”… To advance a more pragmatic scale that would reduce burden, a stepwise procedure of item removal was followed’	Glasgow et al. Pragmatic measures what they are and why we need them. Am J Prev Med. 2013;45(2):237–43 [14].
		“multiple required pragmatic criteria of being important to stakeholders, low in burden for respondents and staff, actionable, and likely sensitive to change. It is also consistent with additional recommended criteria including being broadly applicable, unlikely to cause harm, psychometrically strong, and related to theories or models of implementation”.	
Powell et al. 2017 [7]	Pragmatism not conceptualised in relation to a specific implementation determinant or outcome	“time-consuming or expensive to administer” (antonyms of pragmatic) The 47 criteria previously identified through a systematic literature review and semi-structured interviews [6] were grouped into four categories: acceptable, compatible, easy, and useful. The overarching categories should be helpful in considering the pragmatic construct and have the advantage of parsimony.	Stanick et al. Operationalizing the “pragmatic” measures construct using a stakeholder feedback and a multi-method approach. BMC Health Serv Res. 2018;18(1):882 [6]
		**Acceptable:** (1) Creates a low social desirability bias, (2) Transparent, (3) Acceptable (to staff and clients), (4) Tied to reimbursement, (5) Relevant, (6) Offers relative advantage over existing methods (7) Low cost **Compatible:** (8)Applicable, (9) Efficient, (10) Focused, (11) The output of routine activities, (12) The output of routine activities, (13) Non-duplicative	
		**Easy**: (14) Offers flexible administration time, (15) Easy to interpret, (16) Creates low assessor burden (ease of training, scoring, administration time), (17) Easy to administer, (18) Not wordy, (19) Completed with ease, (20) Requires no expertise, (21) Of low complexity, (22) Uses accessible language, (23) Accessible by phone, (24) Brief, (25) Intuitive, (26) Feasible, (27) Simple, (28) Easy to use, (29) Easy to score, (30) One that offers automated scoring or can be scored elsewhere, (31) Offers a compatible format to setting/user, (32) Low burden	
		**Useful:** (33) Informs decision making, (34) Fits organizational activities, (35) Provides a cut-off score leading to an intervention or treatment plan, (36) Connects to clinical outcomes, (37) Important to clinical care, (38) Produces reliable and valid results, (39) Reveals problems/issues in process or outcomes, (40) Informs adherence of fidelity, (41) Assesses organizational progress over time, (42) Sensitive to change, (43) Meaningful, (44) Confirms efficacy of interventions, (45) Has a meaningful score distribution, (46) Optimizes patient care, (47) Informs clinical intervention selection	
Rye et al. 2017 [26]	Attitudes towards evidence-based practice	“The literature has suggested multiple criteria for ‘pragmatic’ measures including being important to stakeholders; having low burden; being sensitive to change; being broadly applicable; can be used for benchmarking; has norms; is unlikely to cause harm; is psychometrically strong; and is related to theory or model.”	Lewis et al. Advancing implementation science through measure development and evaluation: a study protocol. Implement Sci. 2015;10:102 [30]. Glasgow et al. Pragmatic measures what they are and why we need them. Am J Prev Med. 2013;45(2):237–43 [14].
Smith et al. 2020 [27]	(1) Leadership climate, (2) beliefs about the upcoming transition, and individuals’ (3) use of and (4) attitudes toward EBPs	“Among other characteristics, pragmatic measures should be salient to both the stakeholders and researchers, be perceived as low burden, and have broad applicability”. “more pragmatic (i.e., more salient, shorter, and with broader application)”	Lewis et al. The Society for Implementation Research Collaboration Instrument Review Project: a methodology to promote rigorous evaluation. Implement Sci. 2015;10:2 [31]. Proctor et al. Measurement issues in dissemination and implementation research. In: Brownson et al. (eds). Dissemination and implementation research in health: Translating research to practice. New York: Oxford University Press; 2012 [32]. Glasgow et al. Pragmatic measures: what they are and why we need them. Am J Prev Med. 2013;45:237–43 [14].
Stanick et al. 2018 [6]	Pragmatism not conceptualised in relation to a specific implementation determinant or outcome	“pragmatic (i.e., practical)” “pragmatic qualities (e.g., length: 2- and 9-item versions are available; cost: free of charge)” “pragmatic qualities (e.g., actionable, sensitive to change)” “proprietary and lengthy” (antonyms of pragmatic) “pragmatic measures are those that are (a) important to stakeholders, (b) low burden for respondents and staff, (c) actionable, and (d) sensitive to change”.	None
		“47 terms/phases related to pragmatic measurement construct: (1) brief (2) connects to clinical outcomes (3) creates low assessor burden (ease of training, scoring, administration time (4) easy to use (5) feasible (6) fits organizational activities (7) important to clinical care (8) informs clinical intervention selection (9)	
		informs decision making (10) low burden (11) low cost (12) meaningful (13) not wordy (14) offers a compatible format to setting/user (15) produces reliable and valid results (16) reveals problems/issues in process or outcomes (17) sensitive to change (18) simple (19) the output of routine activities (20) acceptable (to staff and clients) (21) applicable (22) confirms efficacy of interventions (23) creates a low social desirability bias (24) easy to administer (25) easy to interpret (26) easy to score (27) efficient (28) focused (29)	
		generates data that provides a positive feedback loop (not used for staff punishment (30) has a meaningful score distribution (31) non-duplicative (32) of low complexity (33) offers flexible administration time (34) offers relative advantage over existing methods (35) optimizes patient care (36) provides a cut-off score leading to an intervention or treatment plan (36) relevant (37) accessible by phone (38) assesses organizational progress over time (39) completed with ease (40) informs adherence of fidelity (41) intuitive (42) offer automated scoring or can be scored elsewhere (43) requires no expertise (45) tied to reimbursement (46) transparent (47) uses accessible language”	
Stanick et al. 2021 [8]	Pragmatism not conceptualized in relation to a specific implementation determinant or outcome	Training requirements for using implementation measures may be unclear, require specialized education, be too lengthy, or have a time burden to administer, score, and interpret (opposite of pragmatic) ‘pragmatic qualities in mind (e.g., length: 2- and 9-item versions are available; cost: free)	Stanick et al. Operationalizing the “pragmatic” measures construct using a stakeholder feedback and a multi-method approach. BMC Health Serv Res. 2018;18(1):882 [6].
		The original list of 47 terms (Stanick, 2018) [6] and phrases describing the pragmatic measures construct was reduced to 17, while maintaining the four factor solution emergent from the concept mapping study (Acceptable, Compatible, Easy, Useful (Powell, 2017): (1) Produces reliable and valid results (2) Informs clinical or organizational decision-making (3) Creates a low social desirability bias (4) Relevant (5) Offers relative advantage over existing methods (6) Acceptable (by staff and clients) (7) Low cost (8) Applicable (9) Fits organizational activities (10) Uses accessible language (11) Efficient (12) Feasible (13) Easy to interpret (14) Creates low assessor burden (ease of training, scoring, administration time) (15) Items not wordy (16) Completed with ease (17) Brief.	
		Further refined 17 terms to 11 pragmatic qualities, Useful [*n* = 2 items], Compatible [*n* = 1 items], Acceptable [*n* = 3 items], and Easy [*n* = 5 items]. (1) Acceptable (2) Offers Relative Advantage Over Existing Methods (3) Completed with Ease (4) Appropriate (5) Fits Organizational Activities (6) Informs Clinical or Organizational Decision-Making (7) Cost (8) Uses Accessible Language (9) Assessor Burden (Training) (10) Assessor Burden (Interpretation) (11) Length	
Torres et al. 2020 [28]	Implementation citizenship behavior	“Pragmatic (i.e., reliable, valid, high utility) measures”	None

### Implementation determinants and outcomes

In the following section, we report on the implementation determinants and implementation outcomes, and associated instruments that were the focus of the included articles.

#### Implementation determinants

5 of 8 empirical studies defined pragmatism as a measurement construct in relation to one or more quantitative implementation determinant instruments, including:

- **Implementation leadership**, leader behaviors and actions that actively support effective Evidence-Based Practice (EBP) implementation, measured using the Implementation Leadership Scale (ILS) [24].
- **Implementation intentions**, providers’ intentions to use a specific innovation or evidence-based practice (EBP), measured using the Measure of Innovation-Specific Implementation Intentions (MISII) scale [25].
- **Attitudes toward evidence-based practice**, providers’ attitudes to adopting new practices, measured using the Evidence-based Practice Attitude Scale (EBPAS-36 items) [26]
- **Implementation citizenship behavior**, “the discretionary behavior employees perform to support EBP implementation” measured using the Implementation Citizenship Behavior Scale (ICBS) [28].
- One article defined pragmatism in relation to more than one implementation determinant, including (1) **leadership climate,** (2) **beliefs about the upcoming transition, and individuals’** (3) **use of** and (4) **attitudes toward EBPs**, measured using the (1) Implementation Leadership Scale (ILS), (2) Organizational Change Recipients Belief Scale (OCRBS) (3) Evidence Based Practice Questionnaire (EBPQ) Practice Subscale. (4) Evidence Based Practice Attitudes Scale (EBPAS) Openness Subscale, respectively [27].

3 of the 8 empirical articles [6–8], all produced from the same research group, did not conceptualize pragmatism in relation to a specific implementation determinant or implementation outcome. Instead, these articles sought to conceptualize pragmatism as a measurement construct of implementation measures more generally (i.e., determinants, mechanisms, processes, strategies, and outcomes) [6–8]. Similarly, the single included theoretical article defined pragmatism more widely, in relation to pragmatic models, methods, and measures [29].

### Methods used to define/operationalize pragmatism

In this section, we report on the methods employed to define/conceptualize pragmatism as a measurement construct of implementation determinants and/or implementation outcomes in the reviewed studies.

6 of the 9 articles did not use a specific method to define/conceptualize [24–29]. Rather the authors of these 6 articles defined pragmatism within their studies or described pragmatic qualities of implementation measures, with or without citing relevant literature [24–29].

3 of the 9 articles employed specific methods to define/conceptualize pragmatism [6–8]. These 3 articles are linked and report 4 studies that build upon one another. Study 1 used a systematic literature review and stakeholder semi-structured interviews to generate a stakeholder-driven operationalization of pragmatism [6]. Study 2 used concept mapping to further refine the set of criteria identified in Study 1, by identifying conceptually distinct categories of the pragmatic measure construct and providing quantitative ratings of the criteria’s clarity and importance [7]. Concept mapping is a methodology that involves qualitative (e.g., brainstorming) and quantitative (e.g., multidimensional scaling and cluster analysis) techniques to identify relationships between concepts and ideas [33]. Stakeholders with expertise in implementation practice completed a concept mapping activity that involved organizing the pragmatic criteria into conceptually distinct categories and rated their clarity and importance. Study 3 used a Delphi process to achieve consensus among priority pragmatic properties to include in pragmatic rating criteria [8]. Study 4 involved the piloting of the Psychometric and Pragmatic Evidence Rating Scale (PAPERS) pragmatic rating criteria [8].

### Limitations associated with methods used to define/conceptualizing pragmatism

We further summarized the limitations associated with the methods used to define/conceptualize pragmatism as a measurement construct of implementation determinants and outcomes. All limitations reported are those identified by study authors.

All of the articles that employed methods to define/operationalize pragmatism (*n* = 3) highlighted limitations relating to the methods employed [6–8]. These are listed in Table 4.

**Table 4:** Methodological limitations identified by authors employing specific methods to define/conceptualise pragmatism

Author *Methods used to define/conceptualize pragmatism*	Description of methodological limitations identified by authors	Direct quotes detailing methodological limitations
Powell et al. 2017 [7] *Concept mapping*	Ideally stakeholders would have been more extensively engaged in the initial compilation of the pragmatic construct. Issues identified with “concept mapping” as a technique to unpack relationships between various indicators of pragmatism.	“[I]t is possible that engaging our 24 stakeholders in an open process of brainstorming could have yielded a more comprehensive list of potential criteria for the pragmatic construct.” “[W]hile concept mapping provides a rigorous, mixed methods approach to engaging diverse stakeholders and generating conceptual clarity, there are cases in which the way individual items are grouped does not exactly fit with one’s intuitive sense of where they might belong. In some cases when items are located adjacent to a cluster that may provide a better fit, these items can be reassigned as we have done with two of the criteria in this study. In other cases, it is not empirically justified to reassign”.
Stanick et al. 2018 [6] *Systematic literature review* *Semi-structured interviews* Same limitations also highlighted by Stanick et al. 2021 [8]	Systematic literature review limited to published literature Limitations with using the term “pragmatic” to conduct the literature search	“the literature review was subject to bias given that only published literature was assessed; thus, terms related to the pragmatic construct that may fall outside of this source could substantially change the results of the systematic review”. Also, settling on the use of term “pragmatic” to define the construct as it applies to measurement was based on the small, but exiting, literature by Glasgow and colleagues regarding pragmatic measures. It is possible that one of the synonyms identified in the current study may be more broadly suited to define the construct. Given that the extant literature settled on this terminology, however, we continued with it as it seemed the most appropriately suited label for the construct”.

### Stakeholders involved in defining/describing pragmatic qualities of implementation determinant and/or implementation outcomes

#### Diversity and number of stakeholder groups involved

Of the 9 included articles, 3 articles, detailing 4 studies, involved stakeholders in defining/conceptualizing pragmatism [6–8]. Of the 3 articles that involved stakeholders in defining/describing pragmatic qualities, 2 articles recruited mental health professionals (these were Stanick et al. who recruited stakeholders from multiple organization types and service roles i.e.: outpatient community mental health center, school-based mental health, state mental health department, residential center, inpatient hospital (*N* = 7); And Stanick et al. 2021 who recruited implementation leaders from hospitals, and implementation intermediary agency staff (*N* = 26) [6, 8], and 1 article used an international group of stakeholders experienced in behavioral health, including administrators, clinicians and researchers (*N* = 24) [7].

Two of the above articles also involved and engaged a stakeholder panel and/or international advisory board:

- International advisory board (*n* = 9), who were not study participants [7]. The international advisory board vetted the final pragmatic cluster solution and associated labels. Furthermore, a stakeholder panel (*n* = 4), that had been involved in the semi-structured interview study [6], participated in this vetting process.
- International advisory board (*n* = 9), who were not study participants [8]. The international advisory board provided guidance and engaged in refinement activities to determine which pragmatic properties should ultimately be included in the pragmatic measures rating.

### Limitations associated with stakeholders involved

The following limitations are those identified by authors of the included studies. All of the 3 articles that involved stakeholder in the defining/conceptualizing pragmatism, highlighted limitations with the stakeholder groups involved [6–8]. Most generally, these articles identified limitations in the number and/or diversity of stakeholders involved.

In terms of diversity, Stanick et al. [6] and Stanick et al. [8] noted that *“all stakeholders included in the studies that led to the development of the pragmatic rating criteria represented mental health contexts”* and *“it is possible that these stakeholders have very different perspectives about pragmatic implementation measures than do stakeholders from other fields or health more broadly”*. Furthermore, Powell et al. [7] noted that all of their sample worked in behavioral health and specifically noted that their sample did not include policy makers, who may have rated these criteria differently and that *“including a larger sample with more diverse stakeholders would have allowed us to examine whether ratings of importance and clarity differed based upon role or work setting*.” Furthermore, Powell et al. [7] noted that their stakeholder sample primarily included US-based stakeholders and that *“it is possible that a more diverse group would sort and rate these [pragmatic] criteria differently*”. Stanick et al. [6] noted that their international stakeholder group did not include individuals from low- and middle-income countries (LMIC), and *“therefore our representation may be lacking or may impact how the pragmatic construct is defined with respect to measurement in these contexts*”.

In terms of numbers of stakeholders involved, Stanick et al. [6] noted that only 7 stakeholders were recruited to participate in interviews and that if more, and a greater diversity of, stakeholders were recruited it is possible that additional pragmatic criteria may have been identified.

### Evaluation of pragmatic qualities of implementation determinant or implementation outcome measures

The pragmatic qualities (excluding psychometric properties) of implementation determinants and/or implementation outcome measures were evaluated in 1 of the 8 empirical articles included in this review [8]. This article employed the Psychometric and Pragmatic Evidence Rating Scale (PAPERS), which includes eleven properties of pragmatic measures [8]. PAPERS was designed to: “combine pragmatic criteria with psychometric rating criteria, from previous work. […] to inform development of implementation measures and to assess the quality of existing measures.” PAPERS was applied to 60 implementation science measures [8]. Of note, only the objective rating criteria and not the stakeholder-facing criteria, were completed.

Eight articles evaluated the psychometric strength of implementation measures, as a pragmatic quality. Details of the psychometric properties evaluated are presented in Table 5.

**Table 5:** Psychometric properties measured, as a pragmatic quality, across included articles

Authors, date of publication	Implementation determinant and/or outcome instrument	Psychometric properties of implementation determinant and outcome instruments evaluated
Aarons et al. 2016 [24]	The Implementation Leadership Scale (ILS)	• Internal consistency (reliability) • Factorial validity (confirmatory factor analyses) • Convergent and discriminant validity
Moullin et al. 2017 [25]	The Measure of Innovation-Specific Implementation Intentions (MISII)	• Internal consistency (reliability) • Construct validity • Factorial validity (exploratory factor analysis)
Powell et al. 2017 [7]	N/A	Criteria for evaluating pragmatic qualities of implementation measures: (1) produces reliable and valid results, (2) sensitive to change, (3) has a meaningful score distribution
Rye et al. 2017 [26]	The Evidence-based Practice Attitude Scale- 36 (EBPAS-36)	• Internal consistency (reliability) • Cross-cultural validity • Factorial validity (confirmatory factor analyses) • Convergent and discriminant validity
Smith et al. 2020 [27]	Implementation Leadership Scale (ILS) Organizational Change Recipients’ Beliefs Scale (OCRBS) Evidence-Based Practice Questionnaire (EBPQ) Evidence-Based Practice Attitudes Scale (EBPAS)	• Internal consistency (reliability) • Factorial validity (confirmatory factor analysis) • Construct validity
Stanick et al. 2018 [6]	N/A	• Produces reliable and valid results • Sensitive to change • Has a meaningful score distribution
Stanick et al. 2021 [8]	60 implementation determinant and implementation outcome measures	Psychometric properties included in Psychometric and Pragmatic Evidence Rating Scale (PAPERS): • Internal consistency (reliability) • Factorial validity • Construct validity (discriminant, known-groups validity, predictive validity, concurrent) Responsiveness • Norms
Torres et al. 2020 [28]	Implementation citizenship behavior	• Internal consistency (reliability) • Measurement invariance Factorial validity (confirmatory factor analyses) • Construct validity (concurrent)

### Limitations associated with the evaluation of pragmatic qualities of implementation measures, identified by authors

Stanick et al. [8] noted that the rating criteria [PAPERS] they developed was primarily designed to rate self-report implementation measures and *“it remains an empirical question how our PAPERS criteria may respond to different measure formats, such as computer-adapted testing, and future research should consider this.”* [8] Furthermore, Stanick et al. [8] raised concerns *“with the accuracy of measuring pragmatism psychometrically, as a large number of aspects need to be measured in any overall construct, which then become difficult to individually assess: “A […] limitation is that we did not formally assess certain characteristics of the pragmatic rating criteria, such as known-groups validity. Ultimately, what emerged was that the objective criteria appear to have substantial face validity and to be able to assess known groups would primarily mean piloting the criteria; thus, we chose to pilot the criteria with a larger number of measures instead. The rating criteria may be further strengthened in future research focused on establishing these features, or to formally assess to what degree other forms of psychometric properties may be relevant and acceptable (e.g., interrater reliability), establishing cut-off scores for use in various contexts”* [8].

## Discussion

To the best of our knowledge, this is the first review of the literature that focuses on understanding how pragmatism, as a quality and construct of implementation determinant and outcome measures, has been defined, conceptualized and evaluated within the field. This review builds upon and advances previous and noteworthy efforts in the field, by synthesizing the literature and identifying current limitations to the conceptualization and assessment of pragmatic qualities of implementation measures. This work systematically identified limitations relating to (1) the methods that have been used to define/conceptualize pragmatism, (2) the stakeholders that have been involved in defining/conceptualizing pragmatism, and (3) the measures developed to evaluate pragmatism.

### Current state of the field

Despite the importance that has been placed on the identification and development of pragmatic implementation measures, we found very few articles (*n* = 9) that contribute to a cumulative understanding of pragmatism as a quality of quantitative implementation determinant and implementation outcome measures. We found evidence that whilst the term pragmatism is used frequently in the implementation science literature to describe innovations and evidence-based interventions and services, theory and framework application, implementation strategies and study design, it has rarely been used to define/describe the quality of quantitative implementation determinant and outcome measures.

Only three of the included articles engaged and involved stakeholders in defining and conceptualizing pragmatism [6–8]. This represents a major limitation to our current understanding of what makes a measure pragmatic, as Stanick et al. argue *“implementation stakeholders are the ultimate judge—they will use or reject measures based on their perception of whether a measure is pragmatic”* [8]. Of note, we did not include in this review several articles following initial screening on the basis that an implementation determinant or implementation outcome measure was simply refer to/described as “pragmatic” without explicitly defining pragmatism or describing the pragmatic qualities of the measure in question.

#### First recommendation for future research

- Engage and involve stakeholders in defining and conceptualizing pragmatism.

Despite the limited number of articles that define/conceptualize the pragmatic qualities of implementation determinant and implementation outcome measures, we found a degree of overlap in the terms used to describe pragmatic qualities of implementation measures and consensus appears to be emerging concerning what constitutes a pragmatic measure. We identified several descriptors that are frequently used to describe pragmatic qualities of implementation measures, these included “not burdensome”, “brief”, “reliable”, “valid” and “sensitive to change”. It is interesting to note that 3 of the 5 most frequently used terms relate to the psychometric properties of instruments. This point is worthy of further consideration as psychometric and pragmatic properties have been positioned as different constructs/qualities of implementation measures and at the same time psychometric properties have been used to describe the pragmatic quality of implementation measures. Least frequently used descriptors identified included “practical”, “ability to be tailored”, “appropriate”, “length”, “important to stakeholder”, “can be used for benchmarking”, “has norms” and “has high-utility”.

### Limitations in the current evidence base

Although we aimed to provide clarity regarding how pragmatism is conceptualized in relation to implementation measures, we identified significant limitations in the current evidence-base that require further exploration before full conceptual clarity can be reached. Below we discuss the limitations of the current evidence-base and the need, and dangers of not addressing, the identified limitations.

#### Diversity of stakeholders involved in defining and conceptualizing pragmatism

We found limited diversity in the stakeholders involved in defining and conceptualizing pragmatism; with the majority having a background in mental and behavioral health and based in high-income countries—predominantly the USA. Specifically, we did not identify any stakeholders from low and middle-income countries (LMICs) that have contributed to the definition/conceptualization of pragmatism. This is a cause for concern as it has been argued that the availability of pragmatic measures is particularly important for low-resource settings [34]. Furthermore, the stakeholder groups involved in conceptualizing pragmatism have notably not involved consumers (patients, service users, and the public), policy makers, implementation researchers and other applied health researchers that use quantitative implementation measures. This considerably limits the generalizability of findings from the 3 included articles that involved stakeholders in defining and conceptualizing pragmatism [6–8].

#### Second recommendation for future research

- Implement the first recommendation through engaging and involving a wide diversity of stakeholders.

#### Evaluation of pragmatism

We only identified one scale, the Psychometric and Pragmatic Evidence Rating Scale (PAPERS) [8], developed to measure the pragmatic quality, as well as psychometric quality, of implementation determinant and implementation outcome measures. To date, PAPERS has been pilot tested on self-report implementation determinant and implementation outcome measures, as such the utility of PAPERS to assess the pragmatic quality of non-self-reported implementation measures has yet to be tested. A further important point to note is that whilst the initial pilot testing of PAPERS suggests that the pragmatic rating criteria appear to have substantial face validity, further psychometric testing of the scale needs to be undertaken. Furthermore, only the “objective rating criteria” (including cost, uses accessible language, assessor burden (training), assessor burden (interpretation), length) but not the “stakeholder facing criteria” (including acceptable, offers relative advantage over existing methods, completed with ease, appropriate, fits organizational activities, informs clinical or organizational decision-makings) were subjected to pilot testing within the study. Thus, the subjective facing criteria have yet to be tested.

#### Third recommendation for future research

- Subject PAPERS to more rigorous and extensive psychometric evaluation

#### Methods used to define and conceptualize pragmatism

Whilst the methods used to involve stakeholders in defining and conceptualizing pragmatism, identified in the 3 included articles that took this approach, can be considered appropriate, it is not implausible that different methods, and variation in the execution of the methods employed (e.g., if a more comprehensive search strategy in the systematic literature review was employed, more databases were searched) might produce different results in relation to how pragmatism is defined and conceptualized. Indeed, Stanick et al. reported that although significant overlap of terms used to describe pragmatism was found in the literature and stakeholders’ interviews, they identified several terms that were unique to each methodology [6].

#### Fourth recommendation for future research

- Utilize multiple methods to define and conceptualize pragmatism

Last, it is important to note that whilst the three limitations, described above, have been presented as discrete limitations, in reality they are very much interlinked: this is because they are extracted based on 3 articles reporting 4 studies that build upon one another, [6–8]. For example, the pragmatic criteria contained in PAPERS are based on the results of 3 studies conducted with a limited diversity of stakeholders (i.e., mental and behavioral health stakeholders, based in high-income countries). As such, the content of PAPERS might have differed somewhat if a greater diversity of stakeholders were involved in defining and conceptualizing pragmatism.

### Summary of recommendations for future research

Despite the limitations we identified in the current evidence-base, we believe the research conducted to date provides a strong foundation to build upon. Specifically, we recommend that future research involves a wider diversity and number of implementation stakeholders in defining and conceptualizing pragmatism. We believe the preliminary list of 47 pragmatic terms and phases identified by Stanick et al. (2018) [6], and the refined list of 17 pragmatic qualities reported by Stanick et al. [8], are a logical and useful starting point for defining and conceptualizing pragmatism within the field of implementation science. Limitations of this work include lack of diversity of stakeholder involved in defining and conceptualizing pragmatic qualities. Future research should examine whether these terms are transferrable to other implementation stakeholder groups and across a wider diversity of settings and countries. When doing so, we believe it is important that stakeholders are given the opportunity to “add” to the list of terms used to define and conceptualize pragmatism. That is not to say that the other descriptors of pragmatism identified in our scoping review (e.g. unlikely to cause harm, ability to be tailored) should not be subject to further enquire, rather, we suggest that the descriptors identified by Stanick should be prioritized above descriptors not based on empirical research and not involving implementation stakeholders directly. Furthermore, we recommend that future research examines whether the importance of pragmatic criteria differ across stakeholder groups, different settings and countries. Furthermore, we suggest that future research should seek to understand the relative importance of other factors, such as psychometric and methodological strength, that influence the selection of implementation measures. It is unclear whether stakeholders identify several psychometrically strong instruments to potentially use in research or practice, and then select an instrument based on its pragmatic qualities, or whether stakeholders identify several pragmatic instruments and then make a selection based on psychometric strength. Both approaches appear likely but understanding explicitly how stakeholders approach identifying and selecting instruments is important but remains relatively unclear. This implies an inevitable trade-off between psychometric strength and pragmatic quality, which may or may not be the case and remains to be empirically tested. Furthermore, although often referred to as two separate qualities of implementation measures, it is important to again draw attention to the fact that 3 of the 5 most frequently used terms to describe the pragmatic quality of instruments, identified in this review, relate to psychometric properties of instruments (i.e., reliable, valid and sensitive to change’). Last, in terms of measurement of pragmatism, we agree with Stanick et al., that PAPERS needs to be further tested for its psychometric strength, beyond face validity, as well as its utility to non-self-report implementation measures. An important further point to be considered, depending on the results of involving a greater number and diversity of stakeholders, and using different methods: the criteria for evaluating the pragmatic qualities of implementation measures may well need to be modified, rather than finessed. The content domain of pragmatism remains at a very early stage of development and maturity, which invites more developmental research both on PAPERS but also on potential alternative measurement systems.

### The more pragmatic the better?

We found that pragmatic measures are overwhelmingly position as desirable, and whilst we do not disagree that the availability of pragmatic measures is needed, we found very little discussion of the possible negative implications of using highly pragmatic implementation determinant and implementation outcome measures. For example, the use of highly pragmatic measures, might restrict the usefulness of the data yielded in evaluating the success of implementation efforts, comparing the effectiveness of different implementation strategies, and in making decisions about implementation. Similarly, highly pragmatic and generic measures, that are neither context- nor treatment-specific, might be judged to be too generic to be of use both within and outside of the research context. Furthermore, we found little discussion of instances where pragmatic measurement of implementation outcomes and determinants may not be warranted or desirable. Left unsaid this may lead to the assumption that implementation stakeholders should strive, at all times, to identify and use highly pragmatic measures. We found some discussion of how pragmatic methods may not be useful in every study: *“From some perspectives, pragmatic D&I methods are not considered rigorous enough because they do not exert high levels of researcher control to rule out all or most extraneous variables. While this is a defensible position, it is also helpful to remember that one person’s confounders are another researcher’s key independent variables and topic of study.”* [29] Furthermore, it has been suggested that because some pragmatic measures are designed for broad, general use, they may not be a good fit for some local uses or specific conditions [14]. In summary, we found little discussion regarding when pragmatic measures should or should not be used.

### Limitations

Whilst our review has several strengths, it is important to note that it was limited to the published literature in the English language. It is possible that searching a wider grey literature would have identified additional relevant articles. Furthermore, our search strategy could have been more comprehensive. We experimented with several, more comprehensive search strategies. However, the very large number of retrieved articles meant the review was unfeasible to conduct.

## Conclusions

The evidence-base within the implementation instrumentation literature on what pragmatism is and how it might be evaluated is limited. Some of the research identified in this review provides a strong foundation to build upon. Based on the findings of the review, we recommend that future research tests the applicability of current terms used to define and conceptualize the pragmatic qualities of implementation measures in other healthcare settings and countries and among a more diverse group of implementation stakeholders.

## Supplementary Material

ibac064_suppl_Supplementary_File_1
