# Supplementary material for: Defining, conceptualizing and evaluating pragmatic qualities of quantitative instruments measuring implementation determinants and outcomes: a scoping and critical review of the literature and recommendations for future research
Source: Transl Behav Med. 2022 Nov 1;12(11):1049–64. doi: 10.1093/tbm/ibac064 (PMC9677469; doi:10.1093/tbm/ibac064)
Supplement: ibac064_suppl_Supplementary_File_1 [file ibac064_suppl_supplementary_file_1.docx]

**Supplementary File 1: Data Extraction Form**

| **Criterion** | **Data extracted** |
| --- | --- |
| Author, date of publication | Lead author and publication date |
| Country | Country/countries where the study was conducted |
| Study type | Theoretical approach to the topic (e.g., empirical or theoretical) |
| Implementation determinant and/or implementation outcomes of interest | Implementation determinants and/or outcomes that are focused upon in the article (e.g., Implementation leadership scale, Evidence-based Practice Scale-36) |
| Definition of pragmatism and/or terms used to describe pragmatism | Definition of 'pragmatic', including terms associated with pragmatism, as a measurement construct of implementation outcomes (as defined by Proctor's Implementation Outcomes taxonomy) and/or implementation determinants (i.e., factors affecting implementation efforts). |
| Methods used to define/conceptualise pragmatism | 1. Methods used to define/conceptualise pragmatism 2. Limitations relating to the methods used to define/conceptualise pragmatism, identified by study authors |
| Stakeholders engaged/involving in defining/conceptualising pragmatism | 1. Stakeholder group(s) involved in defining and/or describing pragmatism, including stakeholder area of practice and practice area of the study. 2. Limitations relating to the stakeholders involved in defining pragmatism, identified by study authors. |
| Assessment of pragmatism | 1. How pragmatism was assessed 2. Limitations relating to the assessment of pragmatism, identified by study authors. |
